# Encysted Tumor in the Liver: Hydatids

**Published:** 1845-05

**Authors:** 


*Encysted Tumour in the Liver: Hydatids.*—Mr. Walter Freer then exhibited a liver enlarged, and having a very large cyst in its upper and posterior portion containing hydatids.

Thomas Sheldon, aged 36, a miner, previous to this illness, always enjoyed excellent health. August, 1843, was suddenly seized with a severe lancinating pain in the right hypochondrium, which became easier, but did not entirely disappear after the application of leeches, and free evacuation of the intestines. His general health beginning to suffer, and the dull pain in the hypochondrium continuing, he was admitted into the Hospital, January, 1844. On admission he had a firm even tumour, conveying to the fingers an obscure sense of fluctuation, slightly bulging out the ribs behind on the right side, and completely filling the right hypochondrium, reaching down to the iliac region on this side; it extended across the epigastric region into the left hypochondrium, but in the mesial line it did not reach lower than the umbilicus. In February, 1844, he suffered severe pain in the right hypochondrium, and at the painful part a rubbing creaking sound was audible, and by the hand a most distinct fremissiment could be felt. This pain and the physical signs continued till March 20th, when they ceased; the dull pain in the right hypochondrium, which he had felt from the commencement, alone remaining. The natural functions performed almost as in health; pulse generally slow; secretions generally natural; complexion sallow; and extremities emaciated. In April he left the Hospital.

He was re-admitted in the commencement of December, with considerable derangement of the general health; urine scanty; œdematous legs; irregular intermitting action of the heart, and more bulging of the ribs on the right side. The action of the heart became more intermittent and irregular. On the 22d he became delirious, and died twenty-four hours after the supervention of the delirium.

*Autopsy.*—Liver weighed fifteen pounds, the increase being principally confined to the right lobe, which was converted into an enormous cyst, lined with a thick mucous false membrane, and containing some pints of a straw-coloured fluid, in which floated a large number of distinct hydatids, varying in size from a walnut to a pea. The left lobe was not much larger than in health, but presented a nutmeg section. Gall-bladder, natural; heart-cavities, large, with hypertrophied parietes; lungs, healthy; slight pleuritic effusion on the right side. No other morbid appearance in any of the other viscera.—*Prov. Med. & Sur. Journal.*

